# Comparative Molecular Dynamics Simulations of Mitogen-Activated Protein Kinase-Activated Protein Kinase 5

**DOI:** 10.3390/ijms15034878

**Published:** 2014-03-19

**Authors:** Inger Lindin, Yimingjiang Wuxiuer, Aina Westrheim Ravna, Ugo Moens, Ingebrigt Sylte

**Affiliations:** 1 Medical Pharmacology and Toxicology, Department of Medical Biology, Faculty of Health Sciences, UiT the Arctic University of Norway, Tromsø NO-9037, Norway; E-Mails: Inger.Lindin@uit.no (I.L.); Yimingjiang.Wuxiuer@uit.no (Y.W.); aina.w.ravna@uit.no (A.W.R.); 2 Research Group for Molecular Inflammation, Department of Medical Biology, Faculty of Health Sciences, UiT the Arctic University of Norway, Tromsø NO-9037, Norway; E-Mail: ugo.moens@uit.no

**Keywords:** MAPKAPK5, MK5, PRAK, homology modelling, protein-ligand complexes, p38α, protein-protein interactions, molecular dynamics simulations, molecular mechanisms, electrostatic potential surfaces

## Abstract

The mitogen-activated protein kinase-activated protein kinase MK5 is a substrate of the mitogen-activated protein kinases p38, ERK3 and ERK4. Cell culture and animal studies have demonstrated that MK5 is involved in tumour suppression and promotion, embryogenesis, anxiety, cell motility and cell cycle regulation. In the present study, homology models of MK5 were used for molecular dynamics (MD) simulations of: (1) MK5 alone; (2) MK5 in complex with an inhibitor; and (3) MK5 in complex with the interaction partner p38α. The calculations showed that the inhibitor occupied the active site and disrupted the intramolecular network of amino acids. However, intramolecular interactions consistent with an inactive protein kinase fold were not formed. MD with p38α showed that not only the p38 docking region, but also amino acids in the activation segment, αH helix, P-loop, regulatory phosphorylation region and the *C*-terminal of MK5 may be involved in forming a very stable MK5-p38α complex, and that p38α binding decreases the residual fluctuation of the MK5 model. Electrostatic Potential Surface (EPS) calculations of MK5 and p38α showed that electrostatic interactions are important for recognition and binding.

## 1. Introduction

Mammalian mitogen-activated protein kinases (MAPKs) are cytoplasmic protein-Ser/Thr kinases that participate in signal transduction by catalysing the transfer of the γ-phosphoryl group from ATP to a hydroxyl group of the protein substrate. All mammalian cells possess multiple MAPKs involved in controlling cellular processes such as proliferation, differentiation, survival, apoptosis, gene regulation and motility [1].

To date, seven distinct MAPKs pathways have been discovered in mammalian cells, and the MAPKs are divided into conventional and atypical MAPKs. The conventional MAPKs constitute three consecutive phosphorylation events mediated by three Ser/Thr protein kinases. This three-tiered cascade consists of: (1) MAPKs; (2) MAPK kinases that phosphorylate and activate MAPKs; and (3) MAPK kinases kinases that phosphorylate and activate MAPK kinases. Conventional MAPKs include the extracellular signal-regulated kinases 1 and 2 (ERK1/2), p38 MAPKs (p38 α, β, γ, and δ), c-Jun terminal kinases 1-3 (JNK1-3) and the ERK5 [1]. Conventional MAPKs are characterized by a conserved Tyr-X-Tyr motif in the activation loop of the kinase domain. Atypical MAPKs lack this motif, and are not organized in the classical consecutive phosphorylation cascade of three Ser/Thr protein kinases. Atypical MAPKs comprise ERK3/4, ERK7/8 and the Nemo-like kinases (NLK) [2].

Activated MAPKs catalyse the phosphorylation of a large number of proteins including protein kinases, phosphatases, transcription factors and several other proteins. Protein kinases that are phosphorylated by the MAPKs are termed MAPK-activated protein kinases (MAPKAPKs), and so far 11 human Ser/Thr MAPKAPKs have been identified. Based on sequence similarities, MAPKAPKs are subdivided into four groups: (1) Ribosomal S6 kinases (RSK1-4); (2) Mitogen and stress-activated kinases (MSK1 and 2); (3) MAPK-interacting kinases (MNK1 and 2); and (4) MAPKAPK 2, 3 and 5 (MK2, MK3, and MK5) [3,4].

**Figure 1.**
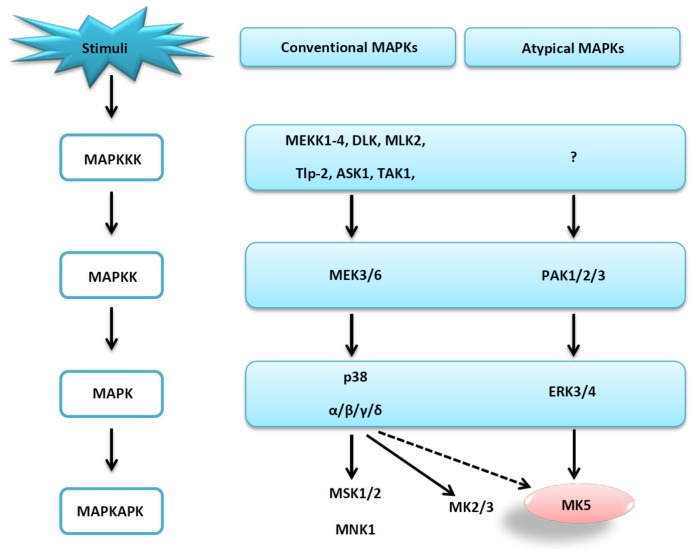
MAPK signalling cascades leading to MK5 activation. Mitogen-activated protein kinase kinase kinases 1-4 (MEKK1-4), the dual leucine zipper kinase (DLK), the mixed lineage kinase (MLK2), the mitogen-activated protein kinase kinase kinase 8 (Tpl-2), the apoptosis signal-regulating kinase 1 (ASK1), the transforming growth factor-β-activated protein kinase (TAK1) and the serine/threonine-protein kinase (TAO1/2) phosphorylates and activates dual specificity mitogen-activated protein kinase kinase3/6 (MEK3/6), which in turn phosphorylates and activates p38 α, β, δ and γ. p38 will then phosphorylate and activate mitogen- and stress-activated protein kinases 1/2 (MSK1/2), the MAPK-interacting kinase 1 (MNK1), MAPK-activated protein kinases 2/3 (MK2/3), and MAPK-activated protein kinase 5 (MK5). In addition to the p38 pathway MK5 can also be activated by extracellular signal-regulated kinases 3/4 (ERK3/4). ERK3/4 are activated by p21-activated kinases 1/2/3 (PAK 1/2/3). The kinase/kinases activating PAK is currently unknown. Dotted line indicates that although activation has been reported, it has to be thoroughly investigated, while solid lines indicate validated activations.

All MAPKAPKs are phosphorylated and activated by different conventional MAPKs. However, MK5 is different from the others since MK5 is phosphorylated and activated both by conventional (p38) and atypical (ERK3 and ERK4) MAPKs (Figure 1) [1,2,5]. ERK3 and ERK4 have been suggested to catalyse phosphorylation at Thr182 and activate MK5 [6,7].

It is generally accepted that MK2 and MK3 are bona fide p38 substrates [4,8]. However, the *in vivo* interaction between p38 and MK5 is under some debate, and is currently not completely resolved (reviewed in [9]). Already in 1998 it was demonstrated that recombinant p38 could phosphorylate MK5 *in vitro* [10]. Thr182 of recombinant MK5 was also found to be phosphorylated by p38α, p38β, p38δ and p38γ *in vitro*, but only p38α and p38β enhanced the intrinsic enzymatic activity of MK5 towards the recombinant small heat shock protein HSP27 [11]. Furthermore, it was demonstrated that p38 regulates the subcellular localization of MK5. Endogenous MK5 and EGFP- or HA-tagged MK5 are predominantly located in the nucleus, but after stress stimulation by TNFα, sorbitol, and sodium arsenite, the p38 pathway is activated and causes nuclear export of MK5 [12,13]. Moreover, experimental studies indicated that nucleocytoplasmic shuttling is controlled through interaction with PKA, Cdc15A and ERK3, ERK4 and p38 [7,12,14–19]. However, yeast two-hybrid assays showed that MK5 interacts only weakly with p38α, and not at all with p38δ and p38γ [6]. Another yeast two-hybrid study identified MK2 as a p38 interaction partner, but not MK5 [7].

MK5 is expressed in all examined cell types and tissues, but most abundantly in heart, brain and hematopoietic progenitors [11,20–22]. Several experimental studies have been performed to elucidate the biological role of MK5. These studies have shown that MK5 may be involved in a wide range of biological processes including control of the cytoskeletal architecture by F-actin remodelling [12,23–26], tumour growth and progression by stimulating angiogenesis [27], tumour suppression by Myc downregulation or antagonizing the JNK pathway [28,29], or interfering with ERK3-mediated cell cycle control, and B-cell development by activating Rag transcription via Foxol [18,30–32]. Further, MK5 was also suggested to be involved in the inflammatory response as a consequence of microbial infection [33] and heart function [2]. Animal studies have also suggested a role of MK5 in neurological processes controlling anxiety and locomotion [34,35]. However, the exact biological role of MK5 is not completely understood, and the main reasons are lack of selective MK5 inhibitors and that MK5 knock-out mice do not display any obvious phenotype under normal conditions [7,36,37].

Structural information about the MK5 and its molecular interactions that can be used to design selective MK5 compounds is therefore of great interest. Regulation of MK5 activity by compounds may also be therapeutically valuable in different disease conditions. The exact three-dimensional structure of MK5 has not been resolved by X-ray crystallography. However, the X-ray crystal structures of MK2 in complex with p38α [38], ADP [39] or small molecular inhibitors [40–43], and of MK3 in complex with an inhibitor [44] are known. Because MK5 has an overall sequence identity of 42% with MK2, and 41% with MK3, it is believed that MK5 may have a 3D fold similar to that of MK2 and MK3.

We have previously constructed 3D homology models of the human analogue of MK5. Our calculations indicated that the models were of high quality and had predictive values [45]. The MK5 models display the protein kinase fold which is well conserved among all members of the protein kinase family. In the present paper, we studied the molecular interactions of MK5 with interaction partners. The program Desmond, as implemented in the Schrödinger suite of programs, was used for comparative molecular dynamics (MD) simulations of MK5. The previously published MK5 homology models were used as starting structures for MD simulations with MK5 alone, in complex with a pyrrolopyrimidone-based inhibitor and in complex with p38α.

## 2. Results and Discussion

### 2.1. The Protein Kinase Fold and Homology Models of MK5

**Figure 2.**
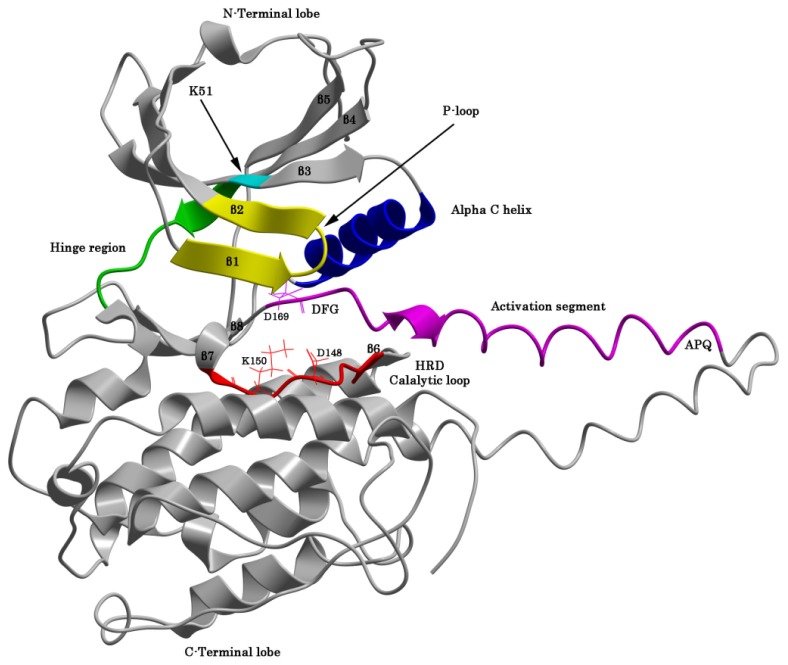
The Cα-trace of the MK5 homology model based on the X-ray structure of MK3 in complex with an inhibitor (PDB id: 3FHR). The construction of the model was described in Lindin *et al.* [45].

The typical bilobal protein kinase fold consists of a small *N*-terminal lobe and large *C*-terminal lobe connected by a structurally flexible linker (hinge) region. Our MK5 models show that this fold is also conserved in MK5 (Figure 2). In MK5, the hinge region corresponds to the segment Glu103-Met104-Met105. The residue *N*-terminal of the hinge region is termed the gatekeeper residue (Met102 in MK5). The X-ray structure of ERK2 shows that the gatekeeper residue confers selectivity for binding nucleotides and small molecular inhibitors, but also seems to control auto-phosphorylation through intramolecular interactions [46]. The *N*-terminal lobe of ERK1 and ERK2 includes a five-stranded antiparallel β-sheet (β1–β5) and a highly conserved αC-helix. The *C*-terminal lobe is mainly α-helical and consists of six conserved α-helixes (αD–αI) and four small β-strands [47]. Figure 2 is showing that the *N*- and *C*-terminal lobes of the MK5 model have an overall organization similar to that of ERK1 and ERK2.

X-ray structures of protein kinases show that the active site is located in between the two lobes and constitutes the binding of ATP and two magnesium ions. The active site is shielded by a loop named the ATP-phosphate binding loop (also called the P-loop). The P-loop is located between β1 and β2 in the N-lobe (Figure 2). The loop contains a glycine-rich segment (GxGxxG), and in ERK1 and ERK2 this loop is suggested to be important for positioning the ATP β- and γ-phosphates for phosphate transfer to the substrate [47]. The GxGxxG domain is also conserved in MK5 and corresponds to the Gly29-Ala30-Gly31-Ile32-Ser33-Gly34. The GAGISG domain is also conserved in MK5 sequences from other species [25].

X-ray structures of ERK1 and ERK2 have shown that the αC-helix of the N-lobe may occur in an activated and inactivated orientation. Structures of inactive and active ERK1 and ERK2 have shown that in the activated structure a glutamic acid of αC forms a salt bridge with the lysine of an AXK sequence in the β3 strand. In MK5, the αC helix consists of residues Pro57-Ala71, and the glutamic acid corresponds to Glu62. The β3 strand is constituted by Arg47-Leu54 in MK5 and the lysine corresponds to the Lys51 of the Ala49-Leu50-Lys51 sequence. The active conformation is often named the αC-in conformation, while the inactive conformation is named the αC-out conformation of protein kinases.

Three amino acids in ERK1 and ERK2 are defining a catalytically important K/D/D motif [47]. These amino acids are also conserved in MK5. In ERK1 and ERK2, the lysine of this motif resides in the β3 strand and corresponds to Lys51 in MK5. In addition to forming a salt bridge with the glutamic acid in αC in the activated state, the lysine also binds the α- and β-phosphorus atoms of ATP. The aspartic acids of the K/D/D motif are located in the *C*-terminal lobe as part of the catalytic loop and the activation segment, respectively (Figure 2). In MK5, the first aspartic acid of this conserved K/D/D motif is part of a HRDLK segment in the catalytic loop, located in the *C*-terminal of the β6-strand, and corresponding to His146-Arg147-Asp148-Leu149-Lys150-Pro151 in MK5. In both ERK1 and ERK2 this aspartic acid is suggested to position the substrate hydroxyl group, and extract the proton of the OH-group and facilitate a nucleophile attack of the substrate oxygen on the γ-phosphorus atom of ATP. The aspartic residue thereby functions as a base in the catalytic reaction [47]. The ERK1 and ERK2 structures also show that the lysine (corresponding to Lys150 in MK5) of this segment binds the oxygen of the substrate hydroxyl group and the γ-phosphorous atom of ATP. The second aspartic acid in the K/D/D motif of ERK1 and ERK2 is the first amino acid in the activation segment that begins with the DFG motif corresponding to Asp169-Phe170-Gly171 in MK5. The DFG motif is followed by the activation loop that contains the regulatory phosphorylation site (Thr182 in MK5). In an active protein kinase, the aspartic acid is pointing into the active site and interacts with two Mg^2+^ ions that interact with the α-, β- and δ phosphates of ATP. The activation loop is fundamental both for substrate binding and catalysis.

In our previous study [45] we constructed several homology models of MK5. In the present study these models were used for MD simulations. The model constructed by using the X-ray structure of MK3 in complex with an inhibitor [44] as template, showed highest accuracy in selecting known MK5 binders in front of decoys during docking. The model based on the MK3 structure was therefore used for 100 ns MD simulation of MK5 alone and for two simulations (100 and 200 ns) of MK5 in complex with a pyrrolopyrimidone-based inhibitor. The MK5 model used for MD with p38α was based on the X-ray crystal structure of MK2 in complex with p38α [38,45].

### 2.2. Stability of the Molecular Systems

High quality X-ray structures are first choice as starting structures for MD simulations and other predictions of molecular mechanisms of action using molecular modelling. However, in the absence of such structures, simulation with high quality homology models may contribute with important structural and mechanistic insight into different classes of proteins and their interactions. Proteins and protein mechanisms that recently have been successfully studied by combining homology modelling and MD simulations include among others: the E7 protein from high- and low-risk types of human papillomavirus [48], the CHeW coupling protein of the chemo taxis signalling complex [49], lipases and their interactions with known inhibitors [50], molecular mechanism of the serotonin transporter [51], conformational changes underlying ion channel opening [52] and molecular mechanism of G-protein-coupled receptors [53–55].

**Figure 3.**
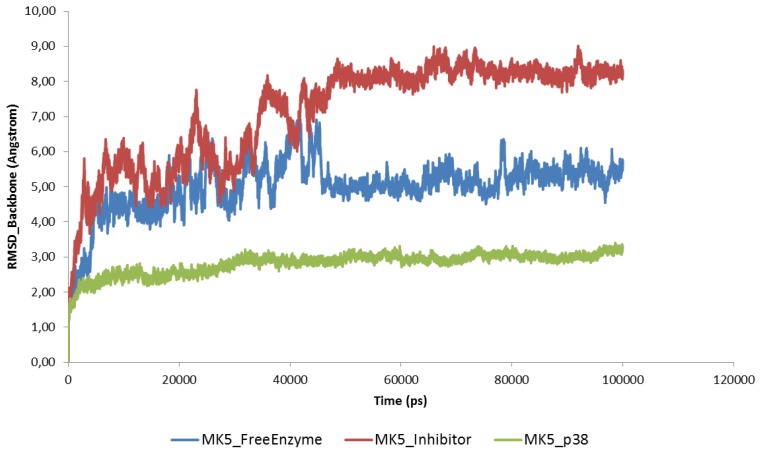
The Root Mean Square Deviations (RMSD) of backbone atoms relative to the starting complexes during 100 ns MD.

**Figure 4.**
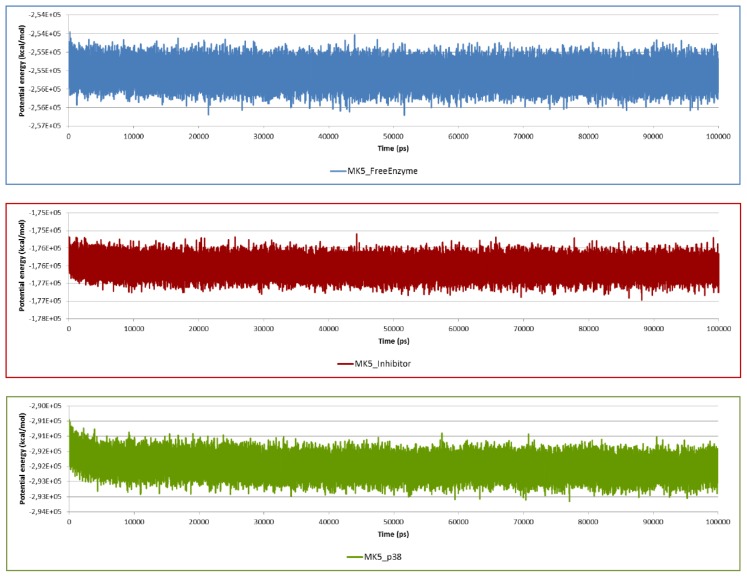
Potential energy of the molecular systems during 100 ns MD simulation.

**Figure 5.**
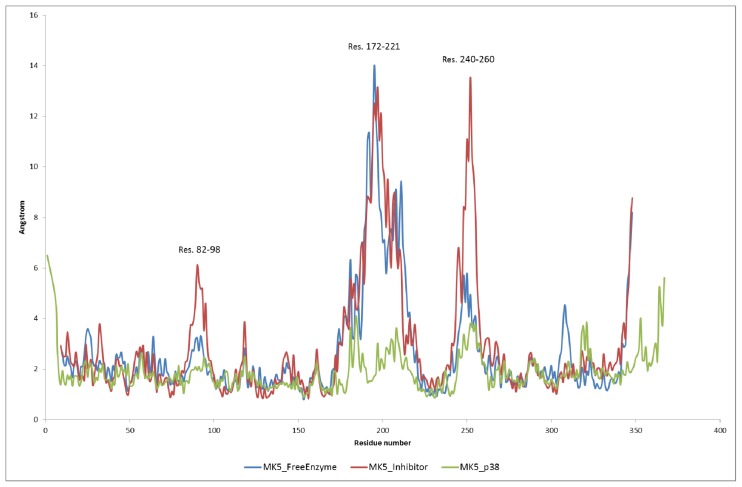
The Root Mean Square Fluctuation (RMSF) during 100 ns MD. All atoms were included in the RMSF calculations.

After using the default relaxation/equilibration protocol of the Desmond program, which includes series of steepest decent energy minimisations and short MD simulations at different temperatures (described in Section 3.3), the molecular systems were simulated for 100 ns. The plots of Root Mean Square Deviations (RMSD) (Figure 3), potential energy (Figure 4) and Root Mean Square Fluctuation (RMSF) (Figure 5) and atomic distance during 100 ns sampling simulations do not include the relaxation/equilibration period. The RMSD of backbone atoms of the MK5 models relative to initial structures show that stable molecular systems were obtained during MD. The RMSD plots indicated that the free MK5 model and the MK5-inhbitor complex were more unstable in the first part of the simulations than the MK5-p38α complex. The template for constructing the MK5 model that was used for MD of the free enzyme and the MK5-inhibitor complexes was a MK3-inhibitor complex (PDB id: 3FHR), and some structural reorganization may be necessary to account for the removal of the MK3 inhibitor during the modelling process. During 100 ns MD with the p38α complex the RMSD of the backbone stabilised at around 3 Å after approximately 30 ns of simulation. During 100 ns MD with the inhibitor, the complex stabilised at around 7.5 Å after about 45 ns, while the free MK5 model stabilised at around 5 Å after about 45 ns of MD (Figure 3). The trend for the 200 ns MD with the inhibitor was similar to the 100 ns MD (data not shown).

The potential energy of the system as the sum of bond, angle, torsion, and non-bonded terms can also be used as a simple measure of system stability. Thus, plots of potential energy as a function of time were generated for the simulated complexes (Figure 4). The plots indicated that all molecular systems were well equilibrated and remained stable throughout 100 ns simulations. This was also observed during a 200 ns MD with the inhibitor (data not shown). The RMSD and the potential energies show that we have obtained structurally and energetically robust models.

### 2.3. MD of the Free MK5 Model

The structurally most flexible regions during 100 ns MD of the native MK5 model were as expected the loop regions, and particularly the region around residues 190–210 (Figure 5). This region is at the C-terminal end of the activation loop. In most protein kinases the activation segment starts with the DFG motif (Asp169-Phe170-Gly171 in MK5) and ends with an APE motif. The APE motif is present in both MK2 and MK3, but in MK5 this motif has been replaced with an APQ motif. The APQ motif seems to be conserved between MK5 from different species. Further, the MK3 structure (PDB: 3FHR) that was the template for constructing the MK5 model is disordered in this part of the activation loop, which may indicate that this part of the activation loop is also structurally flexible in MK3.

In addition, the MK5 has an insert between the APQ motif and the αF-helix compared with MK2 resulting in a longer activation loop than in MK2 and MK3. The lacking part of this loop was constructed by homology with available loop structures in the PDB database [45], and is present at the surface of the MK5 model.

**Figure 6.**
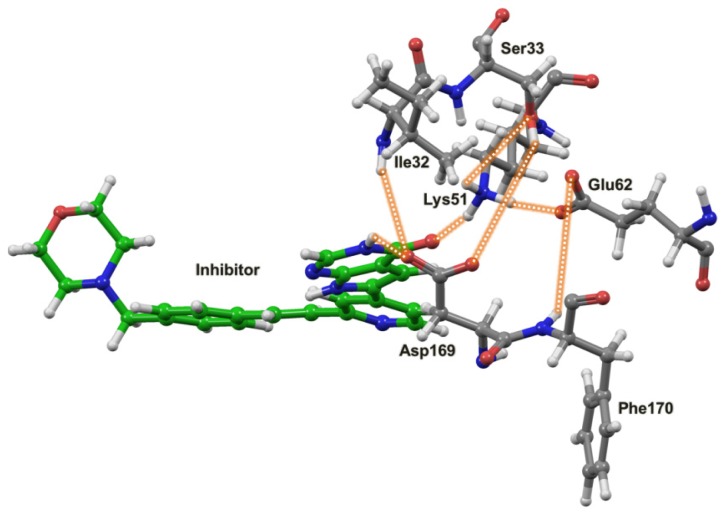
The network of interacting amino acids at the active site of the starting structure for MD with MK5 alone and in complex with the inhibitor. The model was based on an X-ray structure of MK3 in an active kinase conformation, and the MK5 model is therefore also in active kinase conformation. Colour coding of atoms: red; oxygen, blue; nitrogen, green; carbon atoms of the inhibitor, grey; carbon atoms of the amino acids, white; hydrogen atoms. Dotted lines are showing observed interactions between amino acids during MD of the free MK5 and the MK5-inhibitor complex.

**Figure 7.**
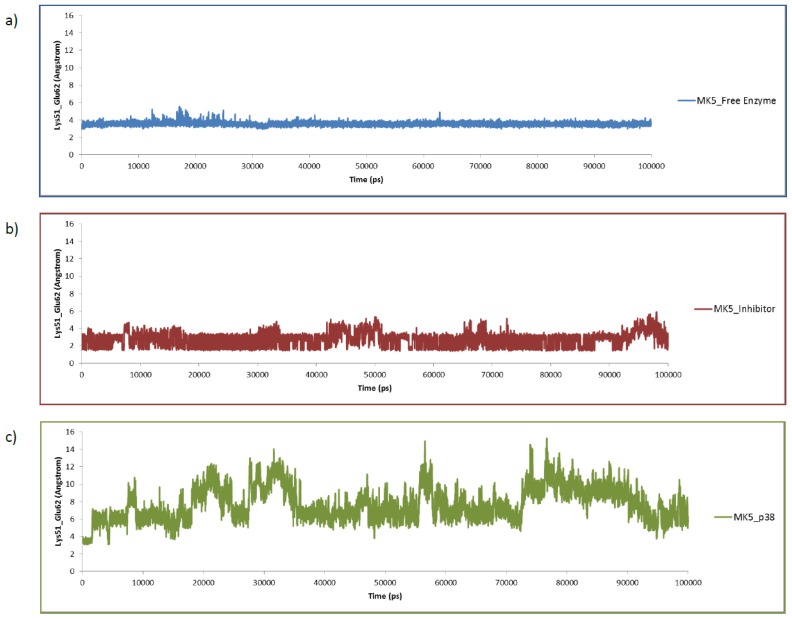
The atomic distances (Å) between Lys51 and Glu62 during 100 ns MD simulations. (**a**) free MK5; (**b**) inhibitor-MK5 complex; (**c**) p38α-MK5 complex.

The same MK5 model was used as starting structure both for the MD of MK5 alone and for the MD of the MK5-inhbitor complex (Figure 6). The starting MK5 model was in an active kinase conformation with interactions between Lys51 and Glu62 (Figures 6 and 7), while Asp169 of the DFG motif in the activation segment interacts with amino acids in the P-loop. The interacting distance between the side chain terminal atoms of Lys51 and Glu62 was varying between 2.5 or 4.0 Å up to 10 ns, then the interacting distance was in the range of 2.5–6 Å, and that lasted up to about 30 ns (Figure 7). After 30 ns, the distance stabilised and was flipping between the range of 1.5–2.3 Å and the range of 2.8–4.0 Å and that lasted up to the end of the simulation. Further, Lys51 also interacted with Ser33 (Figure 6) located in the P-loop. In the beginning of the simulation, the binding distance between the hydroxyl group of Ser33 and Lys51 was around 4 Å, but at the same time as the Lys51-Glu62 atomic distance stabilised (at 30 ns), the Lys51-Ser33 atomic distance (Figure 6) stabilised and was flipping between the range of 1.5–2.3 Å and the range of 2.8–4.0 Å, similar to the Lys51-Glu62 atomic distance. After 30 ns, we observed concerted changes in the interacting distances of the amino acid triplet Ser33, Lys51 and Glu62 (Figure 6). When the atomic distance of Ser33-Lys51 was in the shortest range (1.5–2.3 Å), the atomic distance between Lys51 and Glu62 was also in the shortest range (1.5–2.3 Å), and when Ser33-Lys51 was in the longest range, the Lys51-Glu62 distance was also in the longest range. Glu62 also interacted with backbone NH-group of Phe170 (DFG motif). In the beginning of the MD, the atomic distance varied between 2.0–4 Å. However, at 30 ns when the interactions of Glu62-Lys51 and Lys51-Ser33 stabilised, the interactions between Glu62 and Phe170 also stabilised, and stayed around 2.0 Å for the rest of the MD. Asp169 in the DFG motif, which is coordinating the Mg^2+^ ions and the α and γ phosphate groups when ATP binds, interacted with Ile32 and Ser33 during MD (Figure 6). Asp169 interacted both with the backbone NH-group and the side chain hydroxyl group of Ser33. The Asp169-Ser33 backbone interactions varied in the range of 2.5–4.5 Å up to 33 ns, and then stabilised in the range 1.7–2.3 Å, while Asp169-Ser33 side chain interactions varied between 3–4.5 Å up to 33 ns and then both stabilised in the range of 1.7–2.3 Å for the rest of the MD. The interactions with the backbone NH-group of Ile32 were in the range of 2–3.5 Å up to about 43 ns, but then stabilised at about 2 Å.

The intermolecular network of interactions, including Ser33, Lys51, Glu62 and Ph170, stabilised at around 30 ns, while the interactions of Asp169 stabilised from between 30 and 40 ns of MD. For the rest of the MD, the interactions of Asp169 with Ser33 and the interactions of Phe170 with Glu62 were in the region of 1.7–2.5 Å, while the Lys51-Ser33 and Lys51-Glu62 interactions were flipping between 1.5–2.3 and 2.8–4.0 Å. Taken together, the MD simulation of the free MK5 model indicated that the hydrogen bonding network between amino acids at the active site, consistent with an active open protein kinase fold, was stably formed after about 30 ns of MD and was preserved throughout the MD.

### 2.4. The MK5-Inhibitor Complex

**Figure 8.**
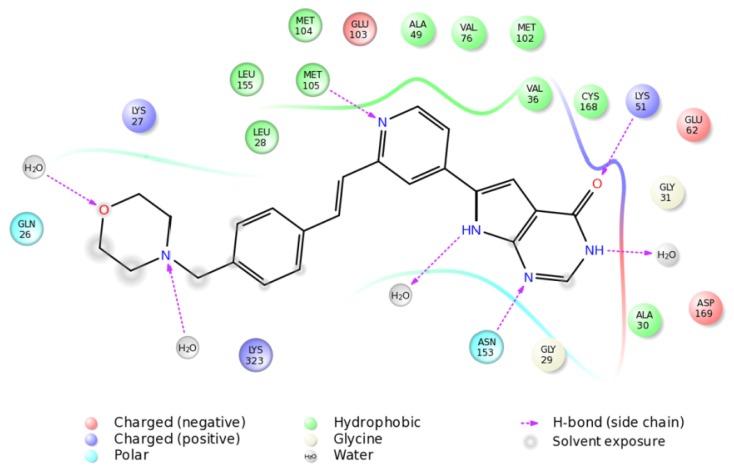
2D plot of the stable inhibitor binding pose observed during the 100 ns MD. Amino acids and water molecules within 4 Å from the inhibitor are included in the figure.

Incorrect ligand protonation may influence ligand-protein interactions during MD. However, predicting the pKa value is not straightforward, especially when several ionisable groups exist in the ligand. According to CHEMBEL database (https://www.ebi.ac.uk/chembl/), the pKa of the pyrrolopyrimidone-based inhibitor is 6.58. pKa predictions using the Jaguar program of the Schrödinger suite of programs, and the ICM-software indicated a pKa value in the range of 6.5–6.6. It is therefore highly possible that the majority of the inhibitor molecules are in neutral form when interacting with MK5. However, it is still quite possible that some MK5 molecules may interact in protonated form. The most probable group for being protonated is the nitrogen atom of morpholine ring. Docking of both the neutral (Figures 6 and 8) and protonated forms of the inhibitor showed that the morpholine ring did not participate in direct interactions with MK5, but instead interacts with water molecules on the surface of MK5 (Figure 8). Based on this, we decided to do MD simulations with a neutral form of the inhibitor.

The MD simulation showed that the loops were also the most flexible regions during 100 ns MD with the inhibitor, and the region around residues 190–210 showed the largest fluctuation (Figure 5). Another very flexible segment was Pro90-Arg95, which was even more flexible than during MD with the free MK5 model. This is a part of the loop between β4 and β5 (Figure 2). In MK5, this loop has an insert of 6 amino acids compared to MK2 and MK3. Further, the region around residue 240–260 was also more flexible during MD with the inhibitor than during MDs with the free model, and with p38α. This loop structure is a part of the activation segment and has insertions in MK5 compared to MK2 and MK3.

**Figure 9.**
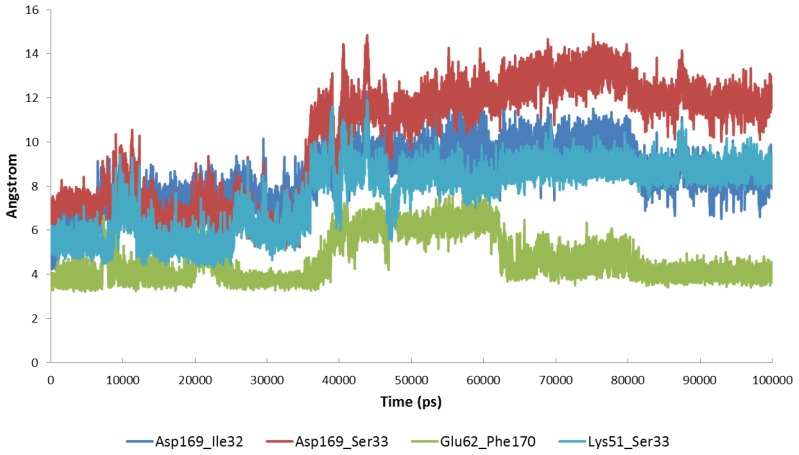
Atom distances between central amino acids at the binding site during 100 ns MD with the inhibitor.

The pyrrollopyrimidone-based inhibitor (Figure 8) was first discovered as a MK2 inhibitor, but was later shown to inhibit MK5 and other protein kinases [56]. The MD simulation indicated that binding of the inhibitor strongly affected the hydrogen bonding network and the dynamics of the active site. The interactions between Asp169 (DFG motif) and Ile32 and Ser33 of the P-loop (Figure 6), that were observed for the free enzyme, were not present during 100 ns MD with inhibitor (Figure 9). The atomic distance between Glu62 and the backbone NH-group of Phe170 that was around 2 Å for most of the MD of the free enzyme was also disturbed by the binding of the inhibitor. The Glu62-Phe170 atomic distance (Figure 6) was around 4 Å up to 40 ns, then increased to 6 Å for the period between 40 and 65 ns, and after 65 ns the atomic distance was around 4 Å (Figure 9). During the MD with the free enzyme, Lys51 formed stable interactions both with Glu62 and with Ser33 in the P-loop (Figure 6). The Lys51-Glu62 interactions were also observed for most of the 100 ns simulation with the inhibitor (Figure 7); however, close binding interactions between Lys51 and Ser33 were not observed (Figure 9). During the 100 ns of MD with inhibitor, a structure with Lys51 in β3 and Glu62 in αC in close proximity, similar to an active protein kinase fold, was obtained, and the interacting distance was varying between 3 and 4 Å for the entire simulation (Figure 7).

The 100 ns MD with the inhibitor indicated that a very stable MK5-inhbitor complex was formed and that the inhibitor was in a stable binding mode throughout the MD. During the 100 ns, the pyrimidine ring system interacted within a charged and polar part of the MK5 binding pocket, and the CO-group of the pyrimidine ring interacted with Lys51 in the αC helix (Figure 8). Up to 25 ns, the interacting distance was in the range of 4–6 Å, but from 25 ns, stable hydrogen bonding interactions (1.8–2.3 Å) were observed. The nitrogen atom of the pyrimidine ring interacted with Asn153, and from 25 ns to the end of the simulation the interacting distance was fluctuating between 1.7 to 4 Å. The pyridine ring interacted in a hydrophobic region of MK5 and the nitrogen interacted with Met105 in the hinge region. Up to 35 ns, the interacting distance was about 2Å, and after 35 ns up to the end of the MD the interacting distance was fluctuating between 2 and 5 Å. The morpholine ring and the phenyl ring interacted with amino acids in the *C*-terminal end of β1 (Gln26-Lys27-Leu28). However, water molecules also seem very important for binding the inhibitor (Figure 8). During MD, water molecules were forming hydrogen bonds with both the oxygen and the nitrogen atom of the morpholine ring, the NH-group of the pyrrolo ring and the NH-group of the pyrimidine ring (Figure 8).

In order to further test the stability of the inhibitor binding mode, we started a new MD simulation of the MK5-inhibitor complex, but now for 200 ns. The 200 ns MD was started from the same equilibrated complex as the 100 ns MD. During the 200 ns, the inhibitor binding mode (data not shown) was similar to the binding mode during the 100 ns. The atomic distances between central amino acids at the active site were also similar to that observed during the 100 ns MD.

Taken together, the simulations with the inhibitor indicated that the inhibitor binds to an active protein kinase fold of MK5 and functions as an inhibitor by occupying the ATP binding site. Upon inhibitor binding, the intramolecular network and dynamical motions of amino acids at the active site are affected (Figures 6 and 8). The intramolecular interactions involving Phe170 (DFG motif) were changed, but the interactions between Lys51 and Glu62 were maintained, indicating that a fully inactive kinase fold was not formed during the MD.

### 2.5. The MK5-p38 Complex

The MK5 protein includes functional binding motifs for the kinases ERK3, ERK4 and p38. Further, experimental studies have indicated that p38 can phosphorylate and activate MK5 and regulate the subcellular location of MK5 [10,11,14,15]. The interactions have been demonstrated *in vitro* after overexpression of MK5 and p38, but the *in vivo* value of these observations is still under some debate, as previously explained, and is not completely resolved [9]. In resting cells, MK5 is predominantly located in the nucleus but is able to shuttle between the nucleus and the cytoplasm. MK5 contains functional nuclear export signals (NES) and nuclear localization signals (NLS), and the opposite action of these motifs may explain the nucleocytoplasmic shuttling of MK5. Activation of the p38 pathway was shown to induce nuclear export of MK5. Both p38α and p38β were reported to control the distinct subcellular localization of MK5 [14,15,57]. Another goal of the present study was therefore to study the molecular interactions between MK5 and p38α.

**Figure 10.**
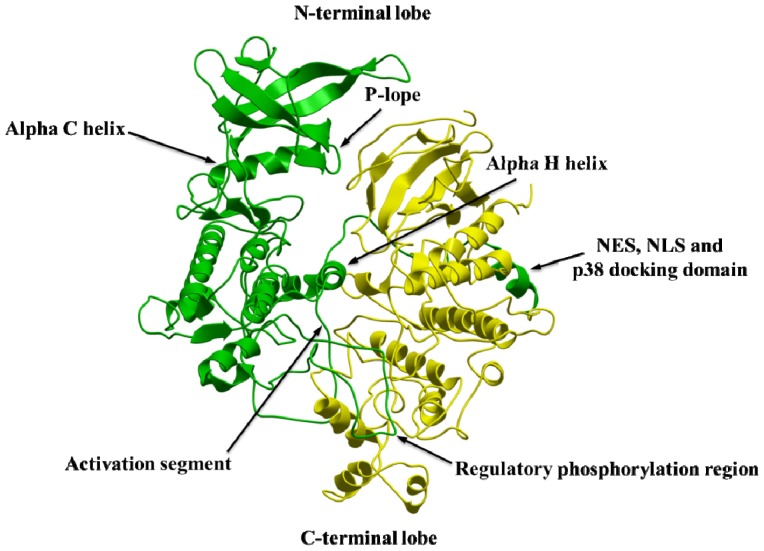
The model of MK5 (green) in complex with p38 (yellow), outlining the important structural elements of MK5 interacting with p38. The Cα-traces of the proteins are shown.

**Figure 11.**
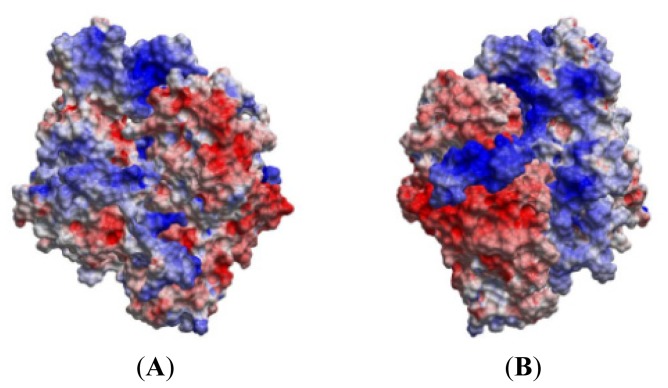

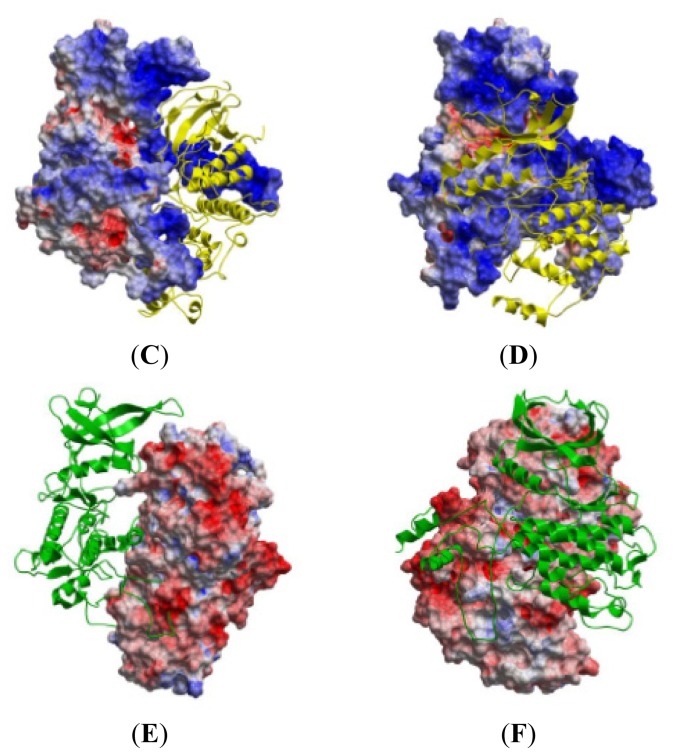
Skin representation of the molecular electrostatic potential surface (EPS) coloured according to the electrostatic potentials. Colour coding: red; ESP < −5 kcal/e.u. charge units, blue; >+5 kcal/e.u. charge units, grey: between −5 and +5 kcal/e.u. charge units. (**A**) Entire complex in the same view as in Figure 8; (**B**) Entire complex rotated 180 degrees (*y*-axis) compared with (**A**); (**C**) The EPS of MK5 with backbone Cα-trace of p38α in yellow. Same view as in (**A**); (**D**) Same as in c, but rotated 90 degrees (*y*-axis) compared with (**C**); (**E**) The EPS of p38α and the backbone Cα-trace of MK5 in green. The same view as in (**A**); (**F**) Same as in (**E**), but rotated 90 degrees (*y*-axis) compared with (**E**).

The MK2 structure in the MK2-p38α X-ray complex (PDB id: 2OZA) was used as a template for constructing the MK5 model of the MK5-p38α complex. The MK5 sequence consists of 473 amino acid residues, while MK2 consists of 400 amino acids, such that based on the template, structure we could not predict the structure of the entire MK5. The MK5 model is therefore *C*-terminally truncated and consists of the amino acids Met7-Gly367. Previous experimental studies have shown that the region Asn356 to Ser373 of MK5 is important for interacting with p38 and was termed the p38 docking site [14]. This indicates that the model did not contain the entire experimentally verified p38 docking site. However, experimental studies have also shown that a *C*-terminal truncated form of MK5 consisting of residues 1 to 368 also binds p38α, although not as strong as the full length MK5 [14]. The docking of p38α into MK5 was guided by the X-ray structure complex of MK2 and p38α. The MK5-p38α complex is depicted in Figure 10. The docked complex showed that not only Asn356-Gly367 in the *C*-terminal of the model is involved in p38α binding, but also other amino acids in the *C*-terminal region are also required (Table 1). In addition, the complex showed that amino acids in the P-loop, activation segment, αH helix, and the regulatory phosphorylation region are also important for binding to p38α (Table 1). The complex showed that MK5 and p38α interact such that the MK5 N-lobe interacts with the p38α N-lobe, and the MK5 C-lobe interacts with p38α C-lobe (Figure 10). The docked complex indicates that the *C*-terminal segment which contains most of the experimentally verified p38α docking site and the regulatory phosphorylation region of MK5 interact (as two arms) at opposite sides of the p38α C-lobe (Figures 10 and 11C).

Calculations of the Electrostatic Potential Surfaces (EPS) indicated that the *C*-terminal segment with the p38 docking site and the regulatory phosphorylation region of MK5 are strongly electropositive and the other regions of MK5 facing p38α are mainly electropositive (Figure 11). However, the ATP binding pocket of MK5 located between the N-lobe and C-lobe is electronegative (Figure 11C). The regions of MK5 facing the solvent are partially electronegative and partially electropositive. p38α has a solvent-exposed side that is mainly electronegative, while the side facing MK5 is mixed electronegative and electropositive with highly electronegative areas in the regions interacting with MK5, the P-loop, helix αH, regulatory phosphorylation site, and p38 docking domain (Figure 11). This observation indicates that the charge distribution on the surface of MK5 and p38α is important for orienting the kinases relative to each other for a proper binding and that the binding is initiated by electrostatic forces.

After optimization and equilibration the complex was simulated for 100 ns. The RMSF during MD (Figure 5) showed that the fluctuations of the flexible loops (including the P-loop) during MD were decreased compared with the simulations with MK5 alone and the inhibitor complexes. However, monitoring the atomic distance between Lys51 and Glu62 at the active site showed that this distance varied a great deal during the MD (Figure 7). A similar trend was also seen for the following atomic distances: Glu62-Phe170, Lys51-Ser33, Asp169-Ser33 and Asp169-Ile32 (data not shown). These atomic distances were very stable during the MD with the free MK5 model. Therefore, it seems like p38α binding not only reduces the structural motions of flexible MK5 loops at the interaction surface, but also destabilizes intramolecular interactions between amino acids in the region of the active site, which may be important for MK5 activation and the binding of substrates to MK5.

**Figure 12.**
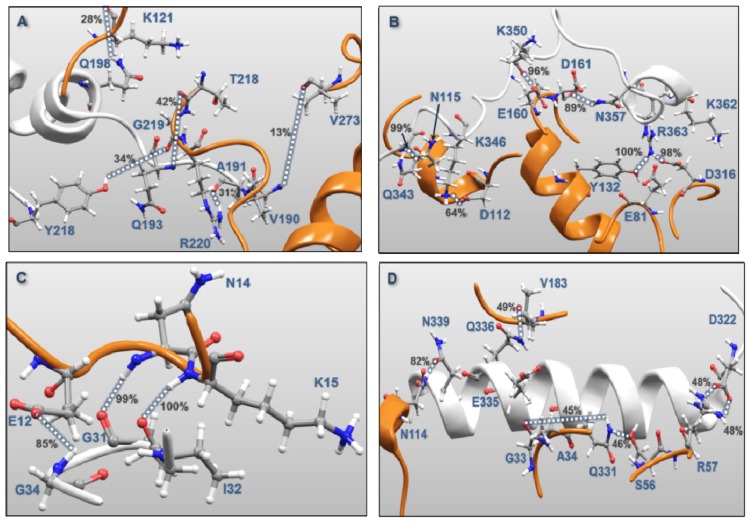

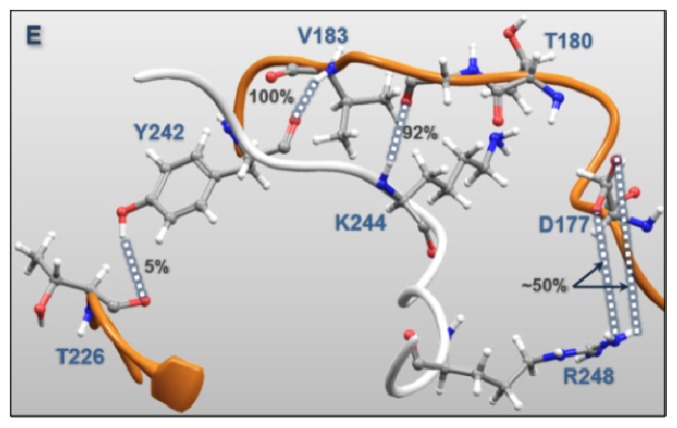
A close up of interacting amino acids forming the MK5-p38α interface. MK5 is in grey ribbon, while p38α is in yellow ribbon. Dashed lines indicate interactions between amino acids in MK5 and p38α. The percentages indicate the percentages of MD frames with the particular interacting atomic distance <3.5 Å. (**A**) the activation segment of MK5; (**B**) the *C*-terminal end of MK5; (**C**) the P-loop of MK5; (**D**) the αH helix of MK5; (**E**) the regulatory phosphorylation region of MK5.

In general, the MK5-p38α complex was structurally very stable throughout the 100 ns MD. During the entire MD, amino acids in the P-loop, activation segment, αH helix, the regulatory phosphorylation region and the *C*-terminal were binding to amino acids of p38α (Figure 12). Table 1 show that amino acids in all these regions of MK5 formed stable interactions with p38α. In the activation segment the region Val190-Gln193 seemed important for binding and formed hydrogen bonds with amino acids in p38α. As previously suggested [14], the *C*-terminal segment was important for binding to p38α, and both Asn357 and Arg363 of the suggested p38 docking site interacted with p38α. During MD, Asn357 formed a hydrogen bond with Asp161, while Arg363 forms a salt bridge with Asp316 and a hydrogen bond with Tyr132 of p38α. Outside the suggested p38 docking site in the *C*-terminal segment, Gln343 formed a hydrogen bond with Asn115 of p38α that was observed in 99.4% of the sampled frames, while Lys346 had a salt bridge with Asp112 that was observed in 63.8% of the frames. In addition Lys350 had strong interactions with both Glu160 and Asp161 (Table 1). In the P-loop, the backbone of Gly31 and Gly34 interacted with the backbone of Lys15 and Glu12, respectively (Table 1). The backbone interaction between Gly31 (P-loop) and Lys15 in p38α was seen in 100% of the frames (Table 1). In αH helix, Asp322 formed salt bridges with Arg57, while Gln331, Gln335, Gln336 and Asn339 also formed stable interactions with p38α. In the regulatory phosphorylation region of MK5, Tyr242, Lys244 and Arg248 formed very stable interactions with p38α and were the most important amino acids for p38α interactions in this part of MK5.

**Figure 13.**
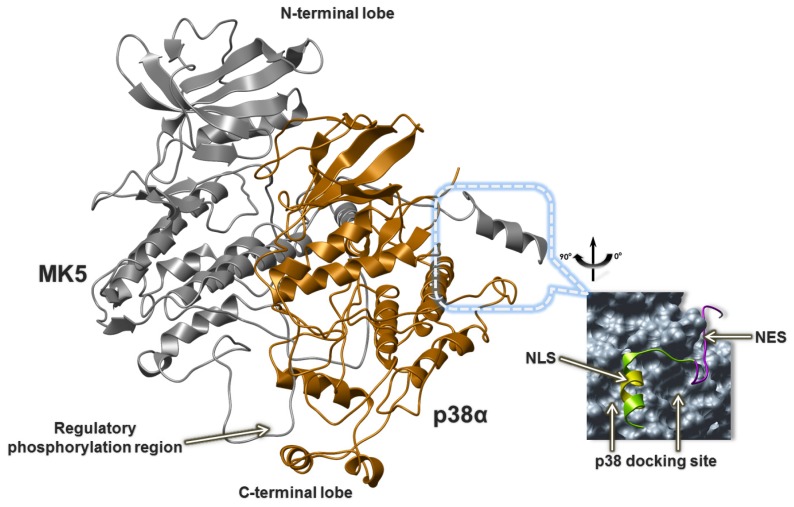
A close up of the nuclear export signals (NES) and nuclear localization signals (NLS) of MK5. The MK5-p38α complex indicates that NES and NLS are masked by the binding of p38α. MK5 in grey ribbon and p38α golden color ribbon.

A combined molecular modelling and site-directed mutagenesis study suggested that Leu156 of p38α is important for directing the MK5-p38α complex to subcellular compartments [57]. When Leu156 was mutated to Val, the complex was mainly localized in the cytoplasm, and it was suggested that Leu156 interacts with the NLS. Our model did not show any direct interaction between Leu156 of p38α and MK5 (Table 1). However, our model showed that mutating Leu156 into Val will have an effect on the 3D structure of p38α, such that the observed interaction of Glu160/Asp161 of p38α with the region around Lys350 of MK5 (Table 1) is disturbed. Lys350 is a part of the NES (Figure 13), and compared to the wild type p38α, the Leu156Val mutant may interact differently with the NES.

## 3. Experimental Section

The MD simulations were performed with the parallel MD program Desmond [58]. The following MD simulations were performed:

1. 100 ns MD of MK5 alone.
2. 100 ns MD of MK5 in complex with a pyrrolopyrimidone-based MK5 inhibitor (Chembel compound id: CHEMBL461139). The inhibitor was in a neutral form.
3. 200 ns MD of MK5 in complex with the same inhibitor. The inhibitor was in a neutral form.
4. 100 ns of MK5 in complex with p38α.

### 3.1. Construction of Initial Complexes

In a previous paper, we constructed homology models MK5 based on different templates using the ICM-software [45]. The models were tested by docking a library of known small molecular inhibitors and decoys. The test docking showed that the MK5 model based on the X-ray structure of MK3 in complex with an inhibitor (PDB id: 3FHR) [44] as template was capable of significantly splitting between binders and decoys. The MK3 X-ray structure is in an active kinase conformation and thereby also the MK5 model. The model consisted of the MK5 residues 9 to 348. The 3FHR-based model was used as a starting structure for MD simulations of MK5 alone and in complex with the inhibitor. The inhibitor was docked into the ATP binding pocket as described in our previous study [45]. In the previous study, we also constructed MK5 models based on the X-ray structure complex of MK2 and p38α (PDB id: 2OZA) [38]. In the present study, this model was used to obtain a starting complex of MK5 and p38 using the 2OZA complex as guide for the p38α docking. This model consisted of the MK5 residues 7 to 367.

### 3.2. Calculation of Electrostatic Potentials

The EPS of the MK5 and p38 were calculated with the Rapid Exact-Boundary Electrostatics (REBEL) method as implemented in the ICM program [59]. This method solves the Poisson equation for a molecule using a boundary element algorithm and generates a 3D surface skin model coloured by electrostatic potential. The potential scale values were set to from −5 to +5 kcal/e.u. charge units. At these scale values, the surface is coloured according the potentials: red potentials <−5, blue potentials >+5. A dielectric constant of 4 was used in the calculations.

### 3.3. Solvation and Refinements

The complexes were optimized with the protein preparation wizard in Maestro V9.1 [60] by assigning bond orders, adding hydrogen and correcting wrong bond types. The molecular systems were neutralized by adding chloride. In Desmond, the volume of space in which the simulation takes place (the global cell) was divided into regular 3D simulation boxes of 10 Å × 10 Å × 10 Å. Each box was assigned to a single Desmond process. These boxes constituted the total simulation space with a total volume of 90 Å × 90 Å × 90 Å. The molecular systems were solvated by SPC (simple point charge) Orthorhombic water box.

All complexes were refined and energy optimized using the default quick relaxation protocol of the Desmond program (D. E. Shaw Research, New York, NY, USA). In short, two rounds of steepest descent minimization were performed with a maximum of 2000 steps with and without restraints (force constant of 50 kcal/mol/Å on all solute atoms). These minimizations were followed by a series of four short MD simulations: (1) 12 ps MD simulation at a temperature of 10 K in the Berendsen NVT ensemble (constant number of particles, volume, and temperature) with solute heavy atoms restrained (force constant of 50 kcal/mol/Å); (2) Twelve ps simulation was performed at 10 K with the same restraints as in (1), but with the Berendsen NPT ensemble (constant number of particles, pressure, and temperature); (3) Using the same restraint as in A and B, a 12 ps simulation was performed in which the temperature was raised to 300 K using the Berendsen NPT ensemble; (4) Finally, a 24 ps simulation at 300 K using the Berendsen NPT ensemble without restraints was used.

### 3.4. The MD Simulations

After the refinements and equilibration steps, MD simulations were performed for all the molecular systems using the OPLS 2005 force field [61]. The pressure was kept constant at one bar and the temperature was kept at 300 K, using the Nose-Hoover chain [62] and Martyna-Tobias-Klein methods [63]. The short-range and long-range Columbic interactions were calculated with a cut off radius of 9 Å and with the Smooth particle mesh method [64] (Ewald tolerance: 1.0 × 10^−9^). The M-SHAKE algorithm was used to constrain bonds containing hydrogen atoms. All the simulations used a multistep RESPA integrator [65] with 2.0 fs time step for bonded interaction and short range non-bonded interactions, and 6.0 fs time step for the long-range non-bonded interactions. During MD, the energies were recorded every 1.2 ps, while the coordinates were recorded every 4.8 ps.

### 3.5. Analysing the Simulations

The simulations were analysed using the Maestro program of the Schrödinger suite of programs. The Root Mean Square Deviations (RMSD) from the initial structure and the root mean square fluctuations (RMSF) were calculated during all MD simulations. All atoms were included in the calculations of the RMSF. Atomic distances important for ligand binding and structural changes in MK5 were calculated for all the simulations.

## 4. Conclusions

In the present study, homology models were used to explore the molecular interactions of MK5 using MD simulations. The homology models have previously been shown to select known MK5 binders in front of decoys during docking [45]. Monitoring energies and RMSD during simulations indicated that energetically and structurally stable complexes were formed. These observations indicate that the homology models were robust and of quality to be used for structural predictions.

The MDs with MK5 alone and with the inhibitor were started from a MK5 conformation in which Lys51 in αC and Glu62 in β3 were in close proximity, which is consistent with an activated protein kinase conformation. This conformation was very stable during 100 ns MD of MK5 alone. During MD simulations with the inhibitor, the atomic distance between Lys51 and Glu62 was also very stable, but the inhibitor binding disrupted the hydrogen bonding network between other amino acids at the binding site and thereby influenced their molecular motions. The inhibitor binding pose was stable both during the 100 ns MD and the 200 ns MD. The pyrrolopyrimidine ring system interacted with a polar and charged region of MK5, including Lys51, while the pyridine ring interacted within a hydrophobic region. In addition, water molecules were forming hydrogen-bonding interactions with the inhibitor during MD (Figure 8). The simulations with the inhibitor indicated that the inhibitor binds to a MK5 conformation consistent with an active protein kinase and thereby functions as an inhibitor by occupying the ATP binding site. The inhibitor binding affects the hydrogen bonding network between active site amino acids, which then increases the atomic distance between Lys51 and Glu62 compared with a fully active protein kinase structure.

Calculations of the ESP of MK5 and p38α showed that the interaction surface of MK5 is strongly electropositive, while the interaction surface of p38α is mainly electronegative, indicating that electrostatic interactions are important for recognition and binding. The MK5-p38α complex shows that the binding of p38α is masking both the NLS and NES of MK5 and that may explain why MK5-p38α complexes are found exclusively in the nucleus [57], which is in agreement with previous suggestions that the NLS is overlapping with the p38 docking site of MK5 [14,57]. The present study shows that p38α can form strong interactions with MK5. During 100 ns MD, both stable hydrogen bonds and salt bridges were observed between the proteins. During the entire MD, amino acids in the P-loop, activation segment, αH helix, the regulatory phosphorylation region and the *C*-terminal were binding to amino acids of p38α. In the design of small molecular compounds that interfere with the binding of p38α to MK5, it may therefore also be important to target central amino acids outside the p38 docking site of MK5 (Table 1) as well as amino acids within the p38 docking site. The present study contributes with increased insight into the structure of MK5 and its interactions with molecular partners. The information can be used in the design of selective compounds interfering with MK5 activity which would be useful in the elucidation of the exact biological role of MK5.

## Figures and Tables

**Table 1.** Interacting pair of amino acids forming at the MK5-p38α interface during 100 ns MD.

MK5		p38α	Percentages of frames with the atomic distance < 3.5 Å	Minimum atomic distance (Å) during 100 ns MD

Location	Amino acid	Amino acid		
**Activation Segment (**Figure 12A)	Val190	Val273	12.8	1.57
	Gln193	Arg220	1.5	1.59
	Gln193	Thr218	41.2	1.55
	Tyr218	Gly219	34.1	1.49
	Gln198	Lys121	28.5	1.56
	Ala191	Arg220	31.1	1.47
***C*-terminal End (**Figure 12B)	Lys362	Glu81	0.7	2.75
	Lys362	Asp316	15.3	1.43
	Arg363	Asp316	0.7	1.61
	Arg363	Asp316	98.2	1.41
	Arg363	Tyr132	99.9	1.48
	Asn357	Asp161	89.3	1.51
	Lys350	Asp161	24.0	1.42
	Lys350	Glu160	96.1	1.50
	Gln343	Asn115	99.4	1.48
	Lys346	Asp112	63.8	1.38
**P-loop (**Figure 12C)	Gly31	Lys15	100.0	1.55
	Ile32	Asn114	99.1	1.53
	Gly34	Glu12	84.7	1.50
**αH helix (**Figure 12D)	Asp322	Arg57	48.4	1.40
	Asp322	Arg57	48.6	1.40
	Gln331	Ser56	46.0	1.57
	Gln331	Gly33	45.3	1.51
	Gln335	Ala34	28.4	1.52
	Gln336	Val183	49.2	1.57
	Asn339	Asn114	81.6	1.52
**Regulatory Phosphorylation Region (**Figure 12E)	Tyr242	Val183	100.0	1.51
	Lys244	Gly181	92.0	1.47
	Tyr242	Thr226	4.8	1.57
	Lys244	Thr180	9.2	1.59
	Arg248	Asp177	49.1	1.39
	Arg248	Asp177	55.4	1.40
